# Long Distance Bicycle Riding Causes Prostate-Specific Antigen to Increase in Men Aged 50 Years and Over

**DOI:** 10.1371/journal.pone.0056030

**Published:** 2013-02-13

**Authors:** Sandra L. Mejak, Julianne Bayliss, Shayne D. Hanks

**Affiliations:** 1 Department of Sports Medicine, Victorian Institute of Sport, Melbourne, Victoria, Australia; 2 Department of Medicine, Monash University, Alfred Hospital, Melbourne, Victoria, Australia; 3 Performance Boost, Melbourne, Victoria, Australia; University Medical Center Rotterdam, The Netherlands

## Abstract

**Objectives:**

To investigate whether bicycle riding alters total prostate-specific antigen (tPSA) serum concentrations in healthy older men.

**Methods:**

129 male participants, ranging in age from 50 to 71 years (mean 55 years), rode in a recreational group bicycle ride of between 55 and 160 kilometers. Blood samples for tPSA analysis were drawn within 60 minutes before starting, and within 5 minutes after completing the ride. The pre-cycling and post-cycling tPSA values were log transformed for normality and compared using paired t-tests. Linear regression was used to assess the relationship between changes in tPSA with age and distance cycled.

**Results:**

Bicycle riding caused tPSA to increase by an average of 9.5% (95% CI = 6.1–12.9; p<0.001) or 0.23 ng/ml. The number of participants with an elevated tPSA (using the standard PSA normal range cut-off of 4.0 ng/ml) increased from two pre-cycle to six post-cycle (or from five to eight when using age-based normal ranges). Univariate linear regression analysis revealed that the change in tPSA was positively correlated with age and the distance cycled.

**Conclusions:**

Cycling causes an average 9.5% increase in tPSA, in healthy male cyclists ≥50 years old, when measured within 5 minutes post cycling. We considered the increase clinically significant as the number of participants with an elevated PSA, according to established cut-offs, increased post-ride. Based on the research published to date, the authors suggest a 24–48 hour period of abstinence from cycling and ejaculation before a PSA test, to avoid spurious results.

## Introduction

Bicycle riding is a common recreational activity, particularly in the older age group for whom joint disease, such as osteoarthritis, often requires a departure from higher impact activities. In Australia, cycling is the fourth most popular sporting activity for males by participant numbers, and eight percent of the male population cycle for physical recreation [1].

Prostate Specific Antigen (PSA) is a glycoprotein secreted by epithelial cells of the normal prostate gland. PSA functions to liquefy the seminal gel at ejaculation [2]. The serum PSA test and digital rectal examination (DRE) are a widely utilised combination of tests used to screen for prostate cancer, although prostate cancer screening guidelines vary widely between countries and different medical organisations. In Australia, screening is usually recommended from the age of 50 years [3]. The PSA test can detect prostate cancer at a point where curative treatment can be offered, but has been noted to have limitations in sensitivity and selectivity in that: the probability of cancer occurring given an elevated PSA is one in three, and prostate cancer can still be present with a normal PSA [4]. Nevertheless, the PSA test remains the most effective screening tool currently available, particularly when it is combined with a DRE.

PSA is produced by both benign and malignant prostate cells, and PSA levels are known to increase in benign prostatic hyperplasia (BPH), prostatitis and cancer [5]. A variety of surgical and non-surgical procedures and activities have been shown to influence PSA. PSA can be transiently elevated by radical prostatectomy (Stamey), transrectal ultrasound-guided needle biopsy [6], [7], and transurethral resection of the prostate (TURP) [6]–[9]. These procedures increase PSA by far greater than 100%, and the PSA can remain elevated for days to weeks. The influence on PSA by these surgical manipulations is probably best summarized by research measuring PSA after radical prostatectomy [8]. There is a large initial increase in PSA after surgery; followed by a decrease in PSA (half life estimated as 2.2+/−0.8 days), and for most patients a period of 14–16 days may be required to allow a fall to normal levels [8], but can occasionally remain elevated for several weeks, particularly when prostate cancer is present.

Non-invasive manipulations, such as ejaculation [10], [11], digital rectal examination (DRE) [12]–[14], and cystoscopy [6], [8], [15] also increase PSA but to a lesser degree, and for shorter periods of time, than surgical manipulations.

Although there is no evidence that general exercise (ie. non-cycling) increases PSA in men with a normal baseline PSA [16], [17], research specifically concentrating on cycling has, however, found evidence that PSA is altered, albeit with conflicting results.

A comprehensive analysis of the 12 previous research reports regarding PSA and cycling, the majority of studies have shown no change to PSA subsequent to cycling [16]–[22], however, three prospective studies [22]–[24] and 2 published case reports [25], [26] have shown an increase in PSA with cycling. The methodologies of these cycling studies varied in many ways from each other, including the age ranges tested, timing of the post-cycling PSA testing, sample size, and the duration and intensity of cycling, and therefore discourage effective comparison. These differences are summarized in Table 1. This lack of consistency prevents definitive conclusions from being drawn in relation to the impact of cycling on PSA.

**Table 1:** Summary of methodology and results of previous studies analyzing the effect of cycling on PSA.

	Age >50 y	Longer distance	Post test within 1 h	PSA Increased with cycling	No change in PSA with cycling
Saka 2009	No	Yes	Yes		X
Hermann 2004	No	Yes	Yes		X
Swain 1997	No (a few>50 y)	Yes	Yes		X
Banfia 1997	No	No	Yes		X
Lippi 2005	No	Not comparable	Not comparable		X
Safford 2006	No/Yes	Yes	No (unclear)	X (≥50 y age group)	X (overall)
Luboldt 2003	Yes	Yes	Yes		X
Kindermann 2011	Yes	Yes	Yes	X	
Oremek 1996	No/Yes	No	Yes	X	

Subsequent to the analysis of previous studies examining the influence of cycling and other prostatic manipulations on PSA, we assessed the effect of cycling by using methods designed to control for the confounding factors that we believed may have led to the findings that cycling did not influence PSA. We aimed to assess whether cycling increased PSA in a large sample of men (n = 129) at an age where prostate screening is commonly conducted (≥50 years), while being representative of typical distances cycled for recreation and fitness (≥55 km), and ensuring that the time elapsed before post-cycling PSA testing was minimized, to eliminate the chance of serum clearance.

## Materials and Methods

### Ethics Statement

The study was approved by the ethics committee of the Australian Institute of Sport in writing on 6^th^ September 2007 (approval number 20070813).

The participants consisted of 129 male cyclists who were invited to take part in the current study as an adjunct to their independent participation in organized and supported recreational group bicycle rides on formed roads. The specified age criteria for selection within the study was 50 years and above. The average age of participants was 55.5 years, with a range of 50–71 years. Potential participants were required to complete a short questionnaire prior to the commencement of the event and based on their responses were either included in or excluded from the study.

Participants were excluded from the study if they had a known history of prostate cancer, benign prostatic hypertrophy, prostatitis, urinary retention, or a history of a prostate operation of any kind. Participants were also excluded if they were on finasteride or other anti-androgen medications, or if they had undertaken a prostate biopsy within the previous 3 months. Participants were not excluded if they had recorded a high PSA during previous routine testing. Participants were advised to abstain from riding on the day of the ride until their pre-cycling blood test was taken.

Sampling took place over five separate recreational one-day cycling events held in Victoria, Australia during 2009 and 2010. The average distance cycled by each participant was 102 km, with a range of 55 km–160 km, with short breaks permitted during the course of the ride. Participants had their pre-ride tPSA taken within 60 minutes before starting the ride, and the post-ride tPSA sample was taken within five minutes of completing the ride. No separate control group was sampled as PSA does not show diurnal variation in patients with or without prostate cancer [27], [28].

Total PSA was measured using a standard test (Centaur analyser, Siemens Healthcare Diagnostics, Tarrytown USA), with an inter-assay coefficient of variation of 5.0%, with all samples analysed at the same central laboratory. An elevation in tPSA was assessed against both the standard PSA normal range cut-off of 4.0 ng/ml, and age-based normal ranges (Table 2), separately. Participants with abnormal results were invited to follow up with their choice of doctor or urologist, or with the urologist involved with the study. Where follow-up information was available it has been commented on in the study. The progress of these participants was followed by the authors via the participants directly and/or their doctors until June 2012, a period ranging from 1 year and 9 months, to 2 years and 8 months post ride.

**Table 2:** Normal ranges for tPSA in ng/ml [37].

Age range (years)	50^th^ percentile (median)	95^th^ percentile (upper limit of normal)
**50–59**	0.85	3.0
**60–69**	1.39	4.0
**70–79**	1.64	5.5

Statistical analysis was conducted using SPSS version 20 for Windows (Chicago, IL, USA). Baseline and follow-up tPSA results were log transformed prior to analysis due to non-normality. Paired t-tests were used to compare pre- versus post-cycling tPSA values. Percentage changes in log transformed serum tPSA represent the absolute differences from baseline multiplied by 100. Linear regression was used to assess the relationship between changes in tPSA with age and distance cycled. All data are presented as means with 95% confidence intervals (CI) or standard deviation (SD) unless otherwise stated. In all instances a p value of 0.05 was considered statistically significant.

## Results

Prior to the ride, the median tPSA value for the study cohort was 0.91 ng/ml, range 0.2 to 13.3 ng/ml. Using the standard PSA normal range cut-off of 4.0 ng/ml, two participants (1.5%) had an elevated tPSA concentration pre-ride. Using age based normal ranges (Table 2), five participants (3.9%) had an elevated tPSA concentration pre-ride.

After the ride, tPSA values increased significantly, by an average of 9.5% (95% CI = 6.1–12.9, p<0.001) or 0.23 ng/ml. Using the standard PSA normal range cut-off of 4.0 ng/ml, six participants (4.7%) had an elevated PSA post-ride. Using age-based normal ranges, eight participants (6.2%) had an elevated tPSA post ride. Only one participant that had an elevated concentration pre-cycling (by age-based normal ranges but not the standard 4.0 ng/ml) subsequently had a normal concentration post-cycling.

**Figure 1:**
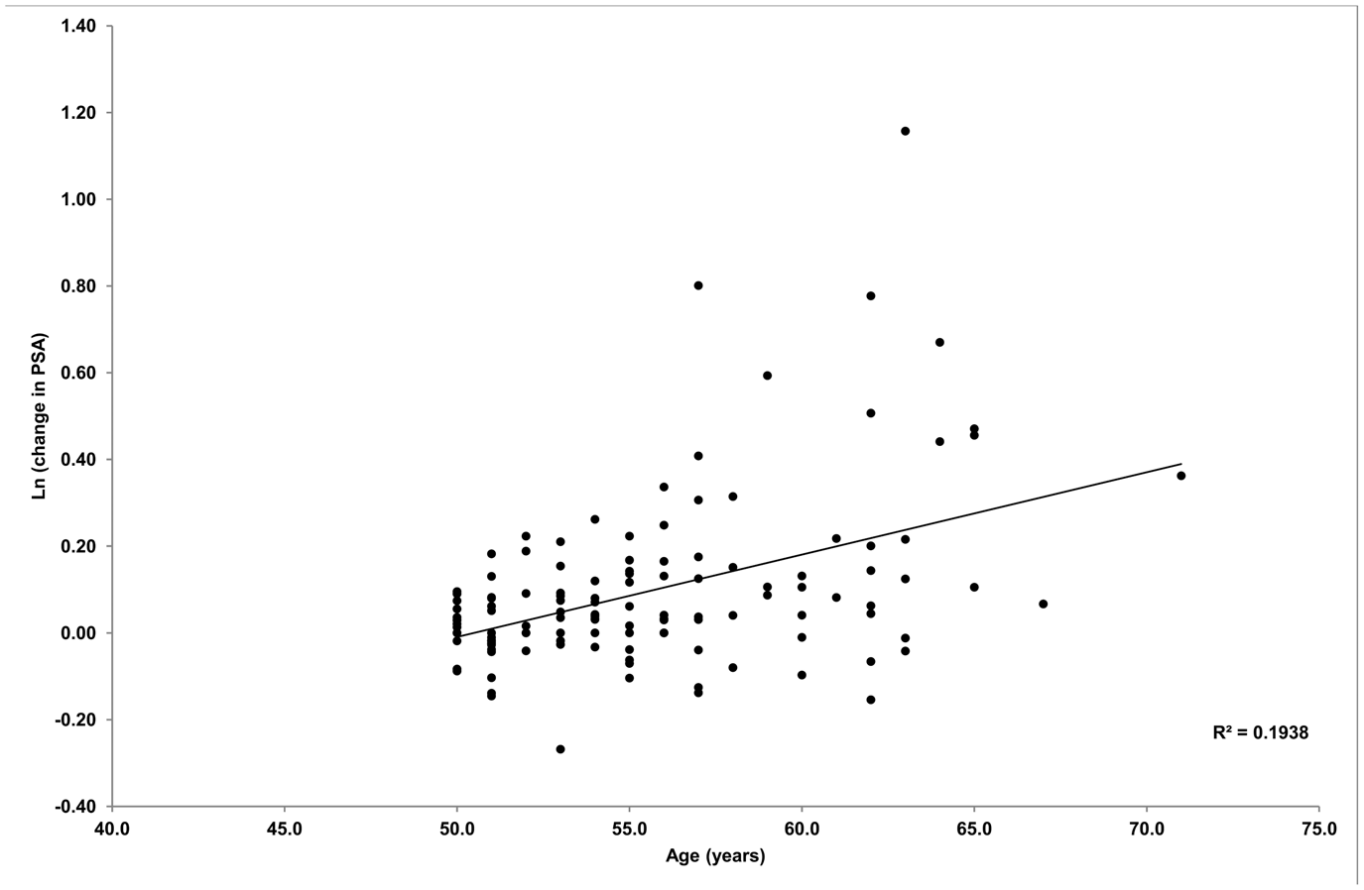
Univariate linear regression of age vs tPSA change (log transformed).

Univariate linear regression analysis revealed that the change in tPSA (log transformed) was positively correlated with both age (r = 0.44, p<0.001) and the distance cycled (r = 0.18, p<0.05). For every single year increase in age, there was a corresponding 1.9% increase in tPSA change (95% CI = 0.012–0.0258, p<0.0001). This is illustrated in Figure 1. Similarly, for every kilometre cycled, the change in tPSA (log transformed) increased by approximately 0.1% (95% CI = 0.000–0.0022, p = 0.04) When both age and distance cycled were entered simultaneously into the model, however, only age remained significantly related to the change in tPSA (p<0.001; model p<0.001; R2 = 0.202) (Table 3).

**Table 3:** Independent risk factors associated with increased tPSA levels.

	Relative Risk	95% confidence interval	p vale
Age	0.018	0.0117–0.0253	<0.0001
Distance cycled	0.0009	−0.0000–0.002	0.07

Model p<0.001; R2 = 0.202).

A summary of the participants with elevated tPSA concentrations, and their clinical follow-up in relation to their PSA and prostate gland, is summarized in Table 4.

**Table 4:** Summary of tPSA results and follow-up, in participants with an elevated tPSA in either the pre- or post-ride test.

Subject age	Pre-ride tPSA	Post-ride tPSA	Follow-up (PSA values = ng/ml)
52 years	3.07†	2.37	After the study was diagnosed with prostatitis, and later BPH (both symptomatic). Medications for BPH cleared all symptoms. PSA since treatment has sat below upper limit of normal.
54 years	3.87†	4.03* †	Symptom free. No further PSA tests done.
54 years	4.66* †	4.51* †	Diagnosed with a urinary tract infection around the time of the testing. Repeat PSA 22 m later 1.29 ng/ml.
57 years	1.36	3.03†	Symptom free. No further PSA tests done.
59 years	3.40†	3.78†	Lost to follow-up.
62 years	13.26* †	22.01* †	5 y prior to the study, elevated tPSA, negative prostate biopsy, and negative MRI. One month post-study: tPSA 9.2 ng/ml with normal MRI but refused biopsy, 18 months post study tPSA 9.6 ng/ml.
62 years	1.94	4.22* †	Symptom free. Repeat PSA 3 m and 2 y later (3.6, 1.8 ng/ml respectively).
63 years	1.60	5.09* †	Symptom-free. Regular PSA tests within normal range.
64 years	2.41	4.71* †	Lost to follow-up

* PSA considered elevated when using the standard PSA normal range cut-off of 4.0 ng/ml.

† PSA considered elevated when using the age-based normal ranges (Table 2).

## Discussion

The purpose of the current study was to determine if long distance cycling increased tPSA in a group of male volunteers ≥50 years old. We found that cycling caused tPSA to increase significantly, by an average of 9.5%, immediately after ceasing cycling, and that the change in tPSA correlated with age and the distance cycled.

In terms of clinical significance, when using the standard PSA normal cut-off range of 4.0 ng/ml, elevated results were recorded in two participants pre-ride and tripled to six participants post-ride. Conversely, when using age based normal ranges, elevated results were recorded in five participants pre-ride and increased to eight participants post-ride. We considered this increase in proportion of elevated PSA results clinically significant, because recording a PSA concentration in the abnormal range may obscure the accuracy of PSA screening for prostate cancer, tipping some individuals into a range where further investigations would be considered appropriate. Any further investigations as a result of artificially elevated PSA may prove unnecessary, expensive, and have adverse physical and psychological impacts on the individual tested.

In examining why the current study design resulted in an increase in PSA when many prior studies have not, we must consider the age of participants, as this appears to be the most important confounding factor from previous designs. Using participants ≥50 years old our study found similar results to the 115 men in the ≥50 years age group of Safford and Crawford [22], who measured PSA subsequent to a four day mountain bike ride. We found a mean increase of 0.23 ng/ml (a 9.5% increase) compared to their result of 0.18 ng/ml which they found was statistically significant (p = 0.0079) but was not believed to be clinically significant, although they did not define what they considered was clinically significant.

Our study also concurs with the findings of Kindermann et al. [24], who tested 21 subjects with an average age of 61±5 years who completed a one hour cycle ergometer activity (they also had a subgroup complete a treadmill test seven days subsequently). They found an increase in tPSA of 25% (1.9±1.7 ng/ml), and free PSA (fPSA) of 92%. The treadmill activity also resulted in a rise in PSA of lesser magnitude. Unique when compared to previous studies, participants in the study by Kindermann et al. [24] were selected on the basis of having a high baseline PSA, without evidence of prostate cancer on biopsy, and this higher baseline seems to have had an impact on the magnitude of the increase and proportion of men demonstrating an increase in PSA.

A closer look at the majority of studies which did not demonstrate a change in PSA after cycling reveals that they sampled men of heterogeneous ages, or sampled only younger men, below the typical age for prostate cancer screening. For example, Saka et al. [20] studied athletes with an average age of 22.4 years (range 16–41 years) and student volunteers with an average age of 24.4 years (range 17–35 years), and tested them before and one hour after a 300 km bicycle ergometer ride. Total PSA, fPSA, and fPSA/tPSA ratio did not change significantly. Similarly, Hermann et al. [19] studied 42 men, with an average age of 35 years (range 29–41 years) before, 15 minutes after, and 3 hours after a 120 km off-road mountain bike race. Free PSA, tPSA, and cPSA were all found not to rise in this study group. Swain et al. [21] studied 20 participants with an average age of 40.7 years (range 27–54 years), riding from 51–162 km, finding no change in PSA. Similarly Banfia et al. [16] studied 12 cyclists with an average age of 22 years on a 24 minute incremental cycle ergometer test and found no change. Safford and Crawford et al. [22] also found no overall change in PSA when they analysed 260 volunteers as a whole. The reason why PSA was found not to change in these studies sampling younger participants may have been because their baseline PSA concentration was lower, and any change may have been less than the interassay coefficient of variation, rendering it not statistically significant.

Luboldt et al [18] tested 33 men with an average age of 61 years (range 50–74 years) before and 1 hour after riding for 90 minutes around a 13 mile (20.8 km) course and concluded that there was no change in both tPSA and fPSA. There was, however, a trend to a rise, but they sampled only 33 men and the result was not statistically significant. This was the only study that sampled men exclusively ≥50 years, without finding an increase in PSA post cycling.

Lippi et al. [17] studied tPSA and fPSA at quarterly intervals, at least 12 hours after exercise, more as a chronic marker. Two cohorts: elite and professional cyclists (average age 27.3 years) and elite cross-country skiers (average age 25.8 years) were individually compared against blood donors (average age 26.7 years) as the control group. No difference in tPSA and fPSA were found between these groups. Being quite a different design to the other cycling studies, it is difficult to postulate the reasons for their results.

Oremek and Seiffert [23] took a group of 301 hospital outpatients of a wide variety of ages (<25 to >70) who cycled on a bicycle ergometer for only 15 minutes, but specifically aimed for pelvic movement to maximize prostate manipulation. Notably these authors found a 2 to 3.3 fold increase in tPSA, fPSA and complexed PSA (cPSA), despite the wide variety of ages sampled, although no study since has demonstrated this magnitude of increase.

The second significant factor that we postulated may have resulted in finding no significant change in PSA with cycling from previous study designs was the interval between cycling and PSA testing. Our study sampled men within 5 minutes of completing their ride, whereas the studies by Saka et al. [20] and Luboldt et al. [18] sampled PSA an hour after completion of the ride.

It may be possible to postulate the duration of elevation of PSA post cycling by examining the study by Kindermann et al [24] in their study consisting of participants with a high baseline PSA. These authors found that for 62% of subjects, the maximum PSA was reached 15 minutes post-exercise (they sampled at 15 minutes, 60 minutes, 120 minutes, 180 minutes, 24 hours, 48 hours, 72 hours and on day 7). Their longer duration of post-cycling testing also found that at 48 hours 10 out of 21 volunteers (48%) had elevated results compared to when resting.

Examining cycling versus a similar prostatic manipulation, Tchetgen et al. [10] and Herschman et al. [11] considered ejaculation’s effect on PSA in men of a typical screening age (≥50 years) and each performed at least 3 post-ejaculation tests from 1 h post to 48 h post ejaculation. In the study by Tchetgen et al, total PSA was found to rise by 41%±4% at 1 hour, then decreased with subsequent tests. Combining the results of Herschman et al. and Tchetgen et al. respectively, at 1 hour 70–86% of men had an increase in tPSA (defined as >15% increase from baseline), 35–20% had an increase at 6 hours, and 40–8% at 24 hours. Herschman et al additionally analysed fPSA and percent free PSA (%fPSA). These parameters showed an increase in a greater proportion of men at 1 hour post ejaculation (100% of men for fPSA and 90% for %fPSA), but fell more rapidly than tPSA (20% having an increased fPSA and 30% having an increased %fPSA at 6 hours, 25% having an increased fPSA and 10% having an increased %fPSA at 24 h). Three studies showed no change [29], a decrease [30], or a decrease followed by an increase [31] in PSA post ejaculation, but had methodology variations such as taking the first sample at a time 24–48 h post ejaculation [29], [30], taking the first sample a wide variety of times post ejaculation [31], testing men well below screening age [30], or performing a DRE in between the first and second tests [31].

Examining another prostatic manipulation, the increase in PSA caused by DRE is usually of the order of 5–10% in studies which start sampling within 1 h after DRE [12], [14], [32]. Ornstein et al [13] showed that 31% and 48% of men had an increase (defined as >15% above baseline) in tPSA and fPSA respectively at 1 h, and that fPSA and tPSA returned to normal in all men by 24 h. Similar modest increases have been shown with flexible cystoscopy [6], [8], [14], [15], [33].

These studies assessing ejaculation, DRE and cystoscopy suggest that non-surgical manipulations of the prostate increase fPSA to a greater extent than complexed PSA (cPSA) (remembering that tPSA is a combination of both fPSA and cPSA). Free PSA and tPSA have been shown to have different half-lives (approximately 2–24 h for fPSA and 2.2–3 days for tPSA) [34]–[36], which may explain why the PSA increase in these non-surgical manipulations has been shown to be short-lived. Our study demonstrated that the manipulation of the prostate caused by cycling affected tPSA to a similar magnitude as ejaculation, DRE and cystoscopy. Extrapolating the current results to take account of those studies and other cycling studies, we assume that cycling is one of those types of manipulations which mostly releases fPSA into the serum. Combining the results of the current study and other studies demonstrating an increase in PSA with cycling, the findings support a period of abstinence of up to 48 h from cycling and possibly more, before a PSA test is taken.

### Conclusions

According to the results of our study, cycling caused an average 9.5% increase in PSA, in healthy male cyclists over 50 years old, when measured within 5 minutes post cycling. This change is statistically and clinically significant. Based on the research published to date, the authors suggest a 24–48 hour period of abstinence from cycling and ejaculation is warranted prior to undertaking a PSA test. An elevated PSA result should also be assessed critically for the recent presence of these activities, and repeated as necessary.
